# HR-Bac, a toolbox based on homologous recombination for expression, screening and production of multiprotein complexes using the baculovirus expression system

**DOI:** 10.1038/s41598-021-04715-5

**Published:** 2022-02-07

**Authors:** Olga Kolesnikova, Amélie Zachayus, Simon Pichard, Judit Osz, Natacha Rochel, Paola Rossolillo, Isabelle Kolb-Cheynel, Nathalie Troffer-Charlier, Emmanuel Compe, Olivier Bensaude, Imre Berger, Arnaud Poterszman

**Affiliations:** 1 Institute of Genetics and of Molecular and Cellular Biology (IGBMC), 1 rue Laurent Fries, Illkirch, France; 2 Centre National de la Recherche Scientifique (CNRS), UMR 7104, Illkirch, France; 3 Institut National de la Santé et de la Recherche Médicale (Inserm), U964, Illkirch, France; 4 Université de Strasbourg, Illkirch, France; 5 Institut de Biologie de l’Ecole Normale Supérieure (IBENS), Ecole Normale Supérieure, CNRS, INSERM, PSL Research University, 46 rue d’Ulm, 75005 Paris, France; 6 Max Planck Bristol Centre for Minimal Biology, Cantock’s Close, University of Bristol, Bristol, BS8 1TS UK; 7 Bristol Synthetic Biology Centre BrisSynBio, School of Biochemistry, 1 Tankard’s Close, University of Bristol, Bristol, BS8 1TD UK; 8 Present Address: EMBL, Heidelberg, Germany

**Keywords:** Expression systems, Biochemistry, Molecular biology

## Abstract

The Baculovirus/insect cell expression system is a powerful technology for reconstitution of eukaryotic macromolecular assemblies. Most multigene expression platforms rely on Tn7-mediated transposition for transferring the expression cassette into the baculoviral genome. This allows a rigorous characterization of recombinant bacmids but involves multiple steps, a limitation when many constructs are to be tested. For parallel expression screening and potential high throughput applications, we have established an open source multigene-expression toolbox exploiting homologous recombination, thus reducing the recombinant baculovirus generation to a single-step procedure and shortening the time from cloning to protein production to 2 weeks. The HR-bac toolbox is composed of a set of engineered bacmids expressing a fluorescent marker to monitor virus propagation and a library of transfer vectors. They contain single or dual expression cassettes bearing different affinity tags and their design facilitates the mix and match utilization of expression units from Multibac constructs. The overall cost of virus generation with HR-bac toolbox is relatively low as the preparation of linearized baculoviral DNA only requires standard reagents. Various multiprotein assemblies (nuclear hormone receptor heterodimers, the P-TEFb or the ternary CAK kinase complex associated with the XPD TFIIH subunit) are used as model systems to validate the toolbox presented.

## Introduction

**Figure 1:**
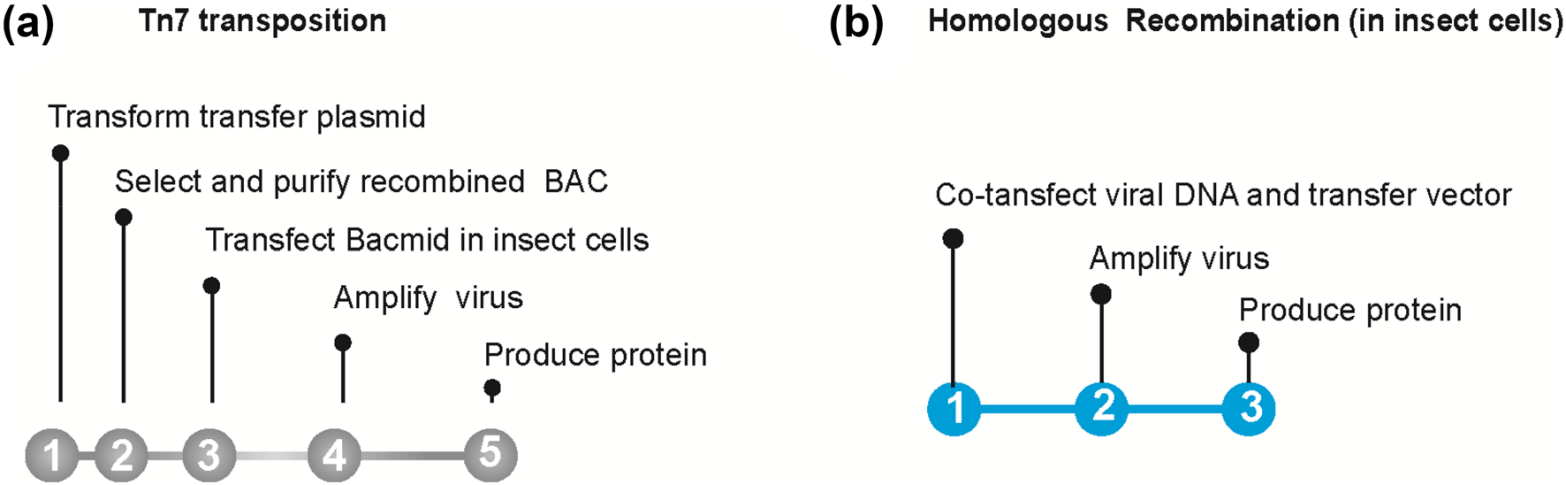
Flowcharts for generation of recombinant viruses and protein production using Tn7-mediated transposition (**a**) or homologous recombination (**b**).

Multiprotein complexes mediate most processes in living systems and have therefore attracted major interest both in fundamental and applied science. As a consequence of their low cellular abundance and heterogeneity, most of them are not readily accessible and must be purified from recombinant sources. The baculovirus expression vector system (BEVS) has emerged at the forefront of heterologous expression of eukaryotic protein complexes overcoming the limitations of bacterial expression systems. BEVS comprise three constituents: the insect host cell line, a baculovirus genome used as a vector and a transfer plasmid(s) bearing gene(s) of interest^1^. Two strategies are in use nowadays for insertion of foreign genes into the baculoviral genome and generation of recombinant viruses. The first strategy is based on the Tn7-mediated transposition of an expression cassette containing the gene(s) of interest into the baculovirus DNA maintained as a BAC in *E. coli* (Fig. 1a). This allows rigorous characterization of the recombinant bacmids in bacteria, before transfection of the viral DNA in insect cells^2^. This approach forms the basis of many available tool-kits for single and multigene expression including the widely used Bac-to-Bac (Invitrogen) and MultiBac (Geneva Biotech)^3^ systems, and others^4–6^. An alternative strategy is based on homologous recombination (HR) between engineered baculoviral DNA and specific transfer vector(s) in insect cells, combining the target gene insertion and recombinant virus generation in a single step (Fig. 1b). Use of an engineered replication-incompetent baculoviral DNA removes a need for purification and further selection of viruses and thus shortens the overall procedure^7–9^. Purified viral DNAs have been commercialized as FlashBAC (OET) or BacMagic (Novagen) and numerous transfer vectors to generate single polypeptide expressing viruses by homologous recombination have been developed during the last decade^9–12^.

We reasoned that homologous recombination, which reduces recombinant baculovirus generation to a single-step procedure represents a highly valuable tool for high-throughput expression screening applications, when expression of many constructs with different affinity tags or mutations need to be tested. HR-based approaches were routinely used for expression screening of isolated proteins. Linearized bacmid DNA are commercially available but the cost can be prohibitive when a large number of viruses need to be generated. Here we describe a baculovirus expression toolbox (HR-Bac) for fast, easy and cost-effective expression screening and production of multiprotein complexes. This toolbox based on traditional homologous recombination consists of two components (Table 1). The first is an engineered baculoviral genome that can replicate in *E. coli* (bacmid) and allows to prepare linearized viral DNA at negligible cost—its preparation only requires standard reagents -. The second is a library of customized transfer vectors containing single or dual expression cassettes bearing different affinity tags to facilitate the purification of the expressed proteins/complexes. The design of these plasmids which contain multiplication modules and LoxP recombination sites facilitates the mix and match utilization of expression units from Multibac constructs^13–15^. To validate the HR-Bac toolbox, we expressed different binary protein complexes including two nuclear hormone receptor heterodimers, the P-TEFb CDK9/cyclinT pair or the ternary CAK kinase complex associated with a fourth protein.

**Table 1:** Components of the HR-Bac toolbox.

Viral DNA	BAC10:KO_1629_, ∆v-cath/chiA	This study
	BAC10:KO_1629_, ∆v-cath/chiA, mCherry	This study
	BAC10:KO_1629_, ∆v-cath/chiA, EGFP	This study
Shuttle vectors	pKI_Ø	This study
	pKI_Fluo (mCherry or EGFP)	This study
Transfer vectors (acceptors)	pAC8 set	^11^
	pAC8_GWs set	This study
	pAC8_MF set	This study
Transfer vectors (donors)	pSPL_pUCDM	^13^
	pSPL-p10/EGPF,-p10/CFP,p10-mCherry	This study
	pIKD, pIDK, pIDC	^14^

## Results

### Engineering of the baculoviral genome

**Figure 2:**
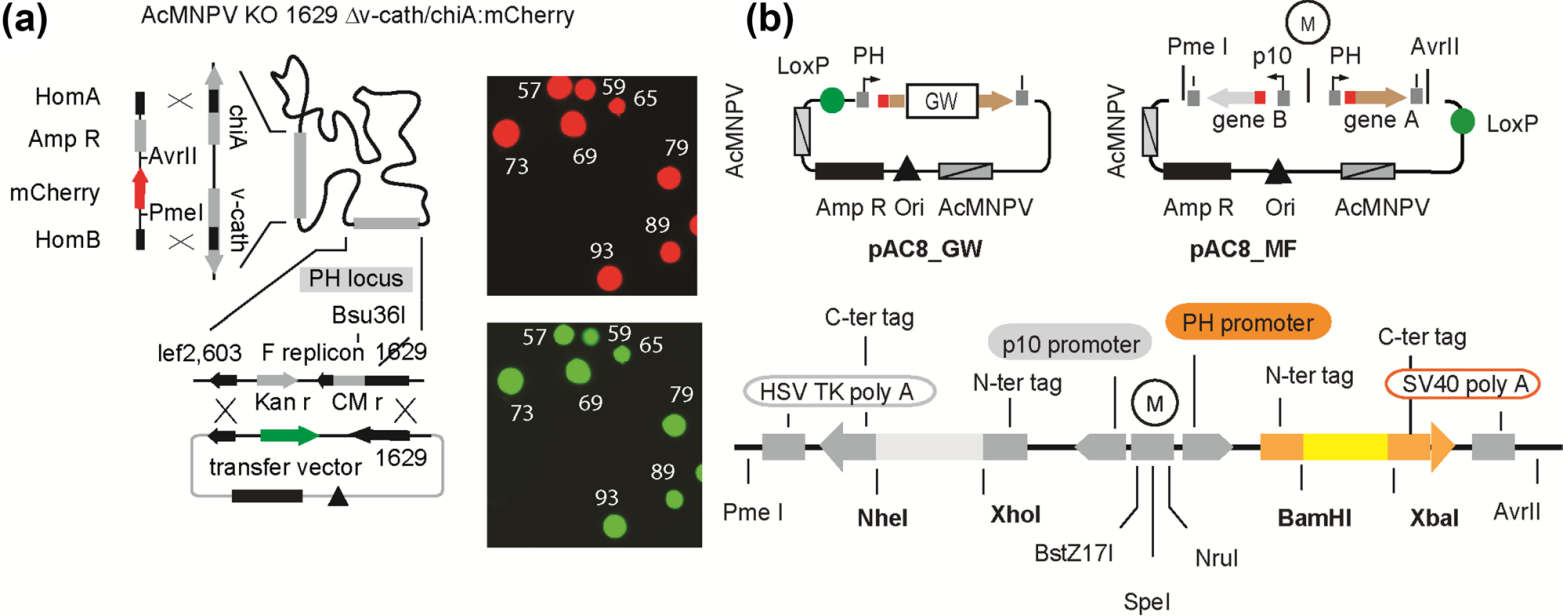
The HR-Bac toolbox components. (**a**) Schematic representation of the AcMNPV BAC10:KO_1629_, Δv-cath/chiA, mCherry bacmid highlighting (i) the modified *v-cath/chiA* locus where the mCherry-coding sequence was inserted to replace *v-cath/chiA* ORFs (left panel), and (ii) the inactivated PH locus in which a part of the essential orf1629 and the polyhedrin coding region were replaced by a bacterial replicon (lower panel). Homologous recombination with the transfer vector bearing the ORF of interest (green arrow) restores the function of orf1629 enabling virus replication and replaces the replicon sequence with the sequence(s) of interest. Sf9 cells infected with a recombinant virus generated using AcMNPV BAC10:KO_1629_ Δv-*cath/chiA*,mCherry viral DNA and a transfer vector containing the EGFP cDNA observed in a microscope (with a ×20 objective); mCherry and EGFP fluorescence for the same field are shown in the upper and lower panels, respectively. (**b**) Schematic representation of pAC8_GW and pAC8_MF transfer vectors. In pAC8_GW, cDNAs are inserted downstream of the polyhedrin (PH) promoter using a Gateway gene insertion cassette (GW). Proteins are expressed in fusion with an N-terminal affinity tag followed by a protease 3C cleavage site (red square) and with a C-terminal c-myc epitope. In pAC8_MF, cDNAs are expressed under the control of divergent PH and p10 promoters. The Ampicillin resistance gene (Amp R, black rectangles), the lef2,603 and orf1629 homology regions (AcMNPV, grey box) and the replication origin (black triangle) are indicated. A detailed map of the pAC8_MF expression cassette is shown in the lower panel. cDNAs are inserted using the BamHI/XbaI and NheI/XhoI restriction sites sequences. The PmeI and AvrII sequences as well as the BstZ17I/SpeI/NruI/ multiplication module from the MultiBac suite (M) are also depicted.

Generation of baculoviruses by homologous recombination is achieved by co-transfecting insect cells with a replication-incompetent viral genome harboring a partial deletion of the orf1629 sequence (HR-bacmid) and a transfer plasmid, which contains the deleted orf1629 sequence. The orf1629 gene encodes the nucleocapsid packaging structural protein (p78/83)^16^ which, although not strictly required, is considered as essential for virus replication. Homologous recombination between the orf1629-defective HR-bacmid and the plasmid restores the capacity of the viral DNA to replicate virus particles and simultaneously inserts ‘the gene under investigation’ into the viral genome^17^. As a source of viral DNA we used an *Autographa californica* multiple nucleopolyhedrovirus (AcMNPV) bacmid (AcMNPV BAC10:KO_1629_) from which a part of the essential orf1629 sequence was deleted^7^ (courtesy of I. Jones). We introduced new modifications in this DNA (Fig. 2a) by first eliminating the v-cath and chiA genes which encode a viral cathepsin-type cysteine protease and a chitinase. Inactivation of these genes was reported to improve maintenance of cellular compartments during infection and to reduce virus-dependent proteolytic activity^13,18,19^. Concomitantly, to monitor the transfection efficiency and propagation of recombinant viruses we introduced the mCherry-coding sequence (resulting bacmid further referred to as AcMNPV BAC10:KO_1629_, Δv-cath/chiA, mCherry) (Suppl. Fig. 1a,b). In addition to the bacmid expressing the mCherry reporter gene, bacmids expressing EGFP or no reporter were also generated (Table 1).

Modified viral DNAs can be easily purified using a plasmid/bacmid isolation kit (a 400 mL culture typically yields 50 μg of purified bacmid that is sufficient for 50 transfections) and offer a time- and cost-effective single step approach to generate multiple recombinant viruses in parallel. To assess the ability of the engineered bacmid to generate recombinant baculoviruses, the viral DNA AcMNPV BAC10:KO_1629_, Δv-cath/chiA, mCherry was purified (Suppl. Fig. 1c) and used to co-transfect Sf9 cells together with a transfer vector encoding the enhanced green fluorescent protein (EGFP) into Sf9 cells. As expected, mCherry and EGFP fluorescence was observed after 5 days incubation, which is not the case in the control experiment where the transfer plasmid encoding EGFP was omitted (Suppl. Fig. 1d). Exponentially growing Sf9 cells infected with the transfection supernatant (V0) stopped dividing and, after 48 h incubation, expressed both mCherry and EGFP (Fig. 2a and Suppl. Fig. 2).

### Design of transfer vectors for multigene expression

A set of transfer vectors originally based on the high copy number pBacPAK8 vector (BD Bioscience) was developed for rapid screening of the expression efficiency and solubility of recombinant proteins (pAC8 plasmids^11,20^). We further adapted this collection for multigene expression. First, a LoxP site, enabling in vitro Cre-mediated recombination with Multibac donor plasmids was inserted, thus providing a flexible platform for generation of transfer vectors for co-expression of multiple proteins (Fig. 2b). Next, to facilitate the use of cDNAs cloned in Gateway Entry vectors—large collections of validated ORFs are now available—pAC8 plasmids were converted into Gateway Destination vectors by inserting a Gateway gene insertion cassette downstream the DNA sequence encoding the N-terminal fusion protein/affinity tag (Suppl. Table 1). Expression clones are created by transferring the genes from Entry clones to pAC8_GWs destination vectors using a standard LR reactions.

We enriched the array of pBacPAK8-derived plasmids with vectors that feature two promoters for expressing two protein simultaneously. These transfer vectors, referred to as pAC8_MF, contain a dual expression cassette with divergent promoters and bear different N- or C-terminal affinity tags for detection or purification (Fig. 2b and Suppl. Table 2). Unique PmeI and AvrII sites as well as the BstZI/SpeI/NruI multiplication module were added to conveniently manipulate complex expression cassettes. Transfer plasmids bearing two dual expression cassettes can be obtained by excising the dual expression cassette of a donor vector by PmeI and AvrII digestion, and inserting it into an acceptor pAC8_MF plasmid via the BstZ17I/SpeI or the NruI/SpeI sites (both pairs PmeI/AvrII compatible) present in the multiplication module (M) (Suppl. Fig. 3a). pAC8_MF vectors also possess a LoxP site and thus can be used as Acceptors in Cre/Lox fusion reaction with any DNA molecule also containing a LoxP site and in particular with the Donor vectors of the MultiBac suite (Suppl. Fig. 3b).

### Validation of the HR-bac pipeline

**Figure 3:**
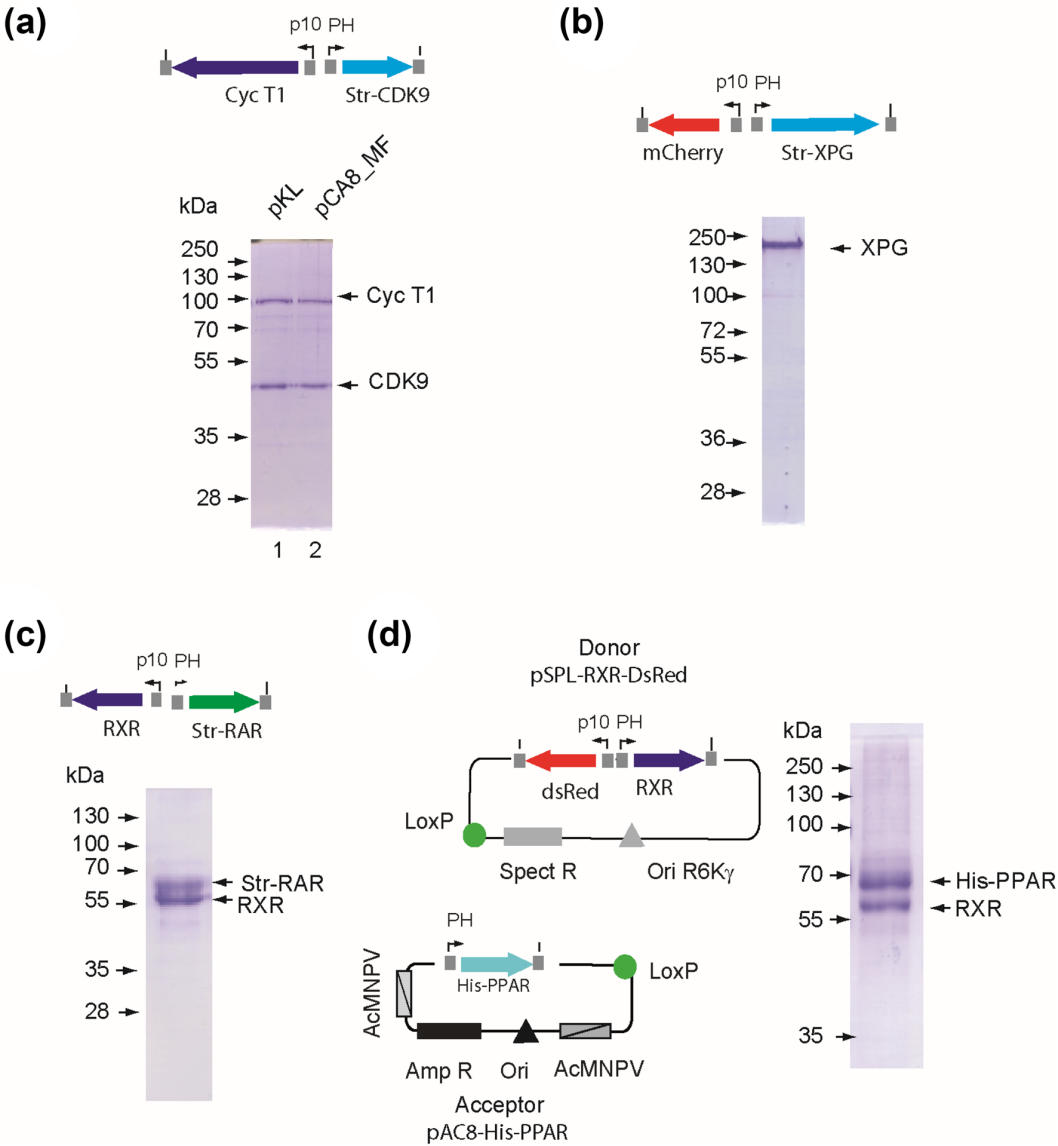
Production of binary complexes. (**a**) CDK9/cyclin T1. The cDNAs encoding Strep-CDK9 and cyclin T1 were cloned into the dual expression cassette of the pKL and pAC8_MF transfer vectors that were used to produce the complex with a virus obtained using the MultiBac toolkit (Tn7 mediated transposition, pK, lane 1) or virus generated with the HR-Bac toolbox (pAC8_MF, lane2). Affinity purified complexes from 10^6^ cells were analyzed using a Coomassie-stained 12.5% SDS gel. (**b**) XPG. Affinity purified Twin-strep tagged *Komagataella phaffii* (Pichia pastoris) XPG homologue produced using the pMF-mCherry/XPG transfer vector and analyzed using a Coomassie-stained 12.5% SDS gel. (**c**) RAR/RXR. Affinity purified Strep-tagged RAR/RXR heterodimer produced using the pMF-RAR/RXR transfer vector analyzed using a Coomassie-stained 12.5% SDS gel. (**d**) Schematic representation of the acceptor vector bearing the His-tagged PPAR coding sequence (light blue) (pAC8-His-PPAR) and the donor vector containing the RXR cDNA (dark blue) under the control of PH promoter. A fusion vector was obtained by Cre-mediated recombination using the lox P sites (green circles) of the acceptor and donor (left panel). Affinity purified His-tagged PPAR/RXR heterodimer produced from the fusion plasmid analyzed on a Coomassie-stained 12.5% SDS gel (right panel). Full-length gels are presented in Suppl. Fig. 6.

**Figure 4:**
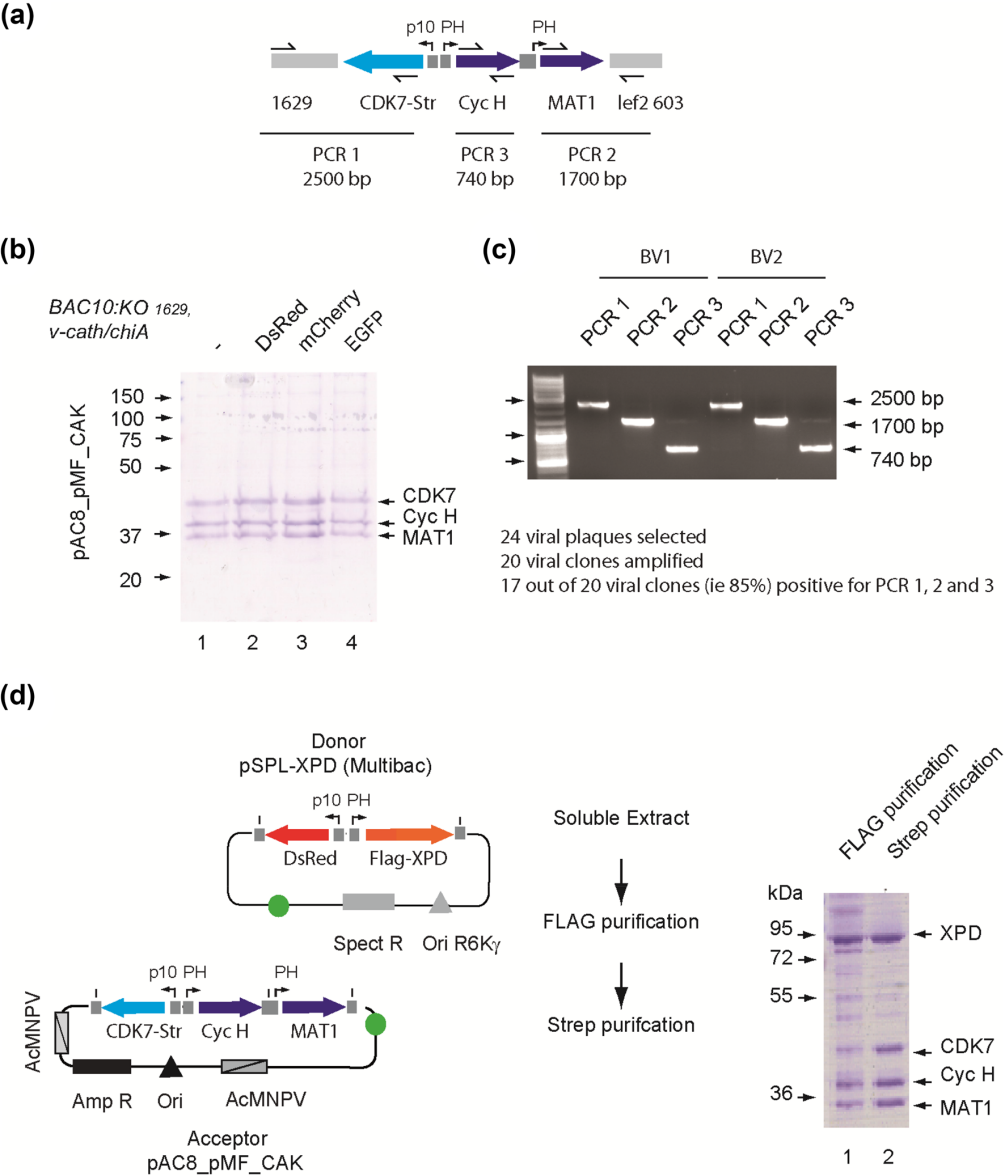
Production of ternary and quaternary complexes. (**a**) Organization of the CAK expression cassette (3900 bp). cDNAs encoding CDK7, cyclin H and MAT1 were assembled into pAC8_MF leading to pAC8_MF_CAK transfer plasmid where CDK7 expression is driven by the p10 promoter and where cyclin H and MAT1 are controlled by the PH promoter. Regions amplified (PCR1, 2500pb; PCR2, 1700pb; PCR3, 700pb) to control the integrity of the construct are also indicated (see **c**). (**b**) The pAC8_MF_CAK plasmid was co-transfected with four modified viral DNAs. Production of CAK from the different viruses was compared (lanes 1,2,3 and 4). (**c**) Baculoviral clones from a CAK expressing virus pool were isolated using plaque purification and amplified. Different PCRs from DNA of 24 viral clones were performed to control the integrity of the construct: PCR1 (2500pb, encompassing the orf1629 sequence and the CDK7 coding sequence), PCR2 (1700 bp, containing the lef2,603 sequence and MAT1 coding sequence) and PCR3 (targeting cyclin H coding sequence). PCR analysis showed that 17 out of the 20 resulting viruses (85%) have the expected complete structure. Here are only presented the 3 different PCR obtained for 2 clones (BV1 and BV2). The PCR products for all the other viral clones tested are shown as Suppl. Fig. 5b. Additionally, the ability of positive and negative baculoviral clones to produce the CAK subunits was evaluated by Western Blots (see Suppl. Fig. 5c). (**d**) Production of the quaternary CAK/XPD complex using one virus coding for four complex subunits. The fusion vector was generated through Lox mediated recombination between the pAC8_MF_CAK acceptor plasmid and the pSPL-DsRed-Flag-XPD donor vector. The complex was purified using FLAG (lane 1) followed by Strep (Lane 2) affinity chromatography. Full-length gels are shown in Suppl. Fig. 6.

To validate the pipeline we selected binary and ternary complexes that were studied in our laboratories. As first model system, we selected the positive transcription elongation factor (P-TEFb) which is composed of the CDK9/cyclin T1 pair and that we previously produced using the MultiBac strategy^21^. The dual expression cassette was transferred from the pKL vector of the Multibac suite^14^ into the pAC8_MF backbone and expression of the corresponding virus generated with the HR-bac pipeline was evaluated. The amount of complex purified from cells infected with the virus obtained by Tn7-mediated transposition (pKL) and by homologous recombination (pAC8_MF) were similar (Fig. 3a). Additional tests of the HR-toolbox included production of a large 120 kDa XPG endonuclease homologue (Fig. 3b), the RAR/RXR or PPAR/RXR nuclear hormone receptor heterodimers (Fig. 3c,d) and the ternary CAK kinase complex (Fig. 4). To produce the nuclear receptor heterodimer RAR/RXR, cDNAs encoding the two polypeptides were cloned under the control of the PH and p10 promoters of the pAC8_MF-PH-TwinStrep-Nter plasmid (Fig. 3c). To produce the PPAR/RXR heterodimer, we assembled a bicistronic transfer vector encoding PPAR and RXR by Cre/LoxP recombination using the pAC8-His-PPAR acceptor plasmid and the pSPL-RXR donor (Fig. 3d). Analysis of the Ni-affinity pool by size exclusion chromatography revealed that PPAR and RXR are present in stoichiometric amounts and the two proteins co-elute as a single symmetrical peak (Suppl. Fig. 4).

The cyclin activating kinase complex (CAK), composed of the CDK7, cyclin H and MAT1 proteins exists as an isolated complex but also is found associated with the XPD helicase or as part of the general transcription/DNA repair factor TFIIH^22^. We routinely express the CDK7/cyclin H pair and the CDK7/cyclin H/MAT1 heterotrimeric complex for structure–function studies using pAC8_MF derivatives^23,24^. For the latter, a 3900 bp expression cassette with divergent p10 and PH promoters drive the production of CDK7 and of cyclin H and MAT1, respectively (Fig. 4a). Here we expressed CAK from viruses obtained by co-transfection of the same transfer plasmid with different AcMNPV bacmids of the HR-bac toolbox (Table 1) to verify that expression of the fluorescent reporter does not interfere with that of the target complex (Fig. 4b). We also performed a plaque assay with a virus pool used for the production of CAK to estimate the success rate of the recombination process. Baculoviral clones from the virus pool were isolated by plaque purification and analyzed by PCR: 17 out of the 20 resulting viruses (85%) had the expected complete structure (Fig. 4c and Suppl. Fig. 5b). Finally, to produce the four-subunit complex CAK/XPD, a transfer vector coding for all four subunits was obtained by Cre/Lox recombination between pAC8_pMF_CAK and pSPL-XPD. Using the corresponding virus full CAK/XPD complex was produced (Fig. 4d). As XPD specifically interacts with the MAT1 subunit of CAK and as the expression level of CDK7 is lower than that of Cyc H and MAT1 (Illustrated Suppl. Fig. 5c), tandem affinity purification was required to remove the excess of MAT1/XPD complex.

## Discussion

**Figure 5:**
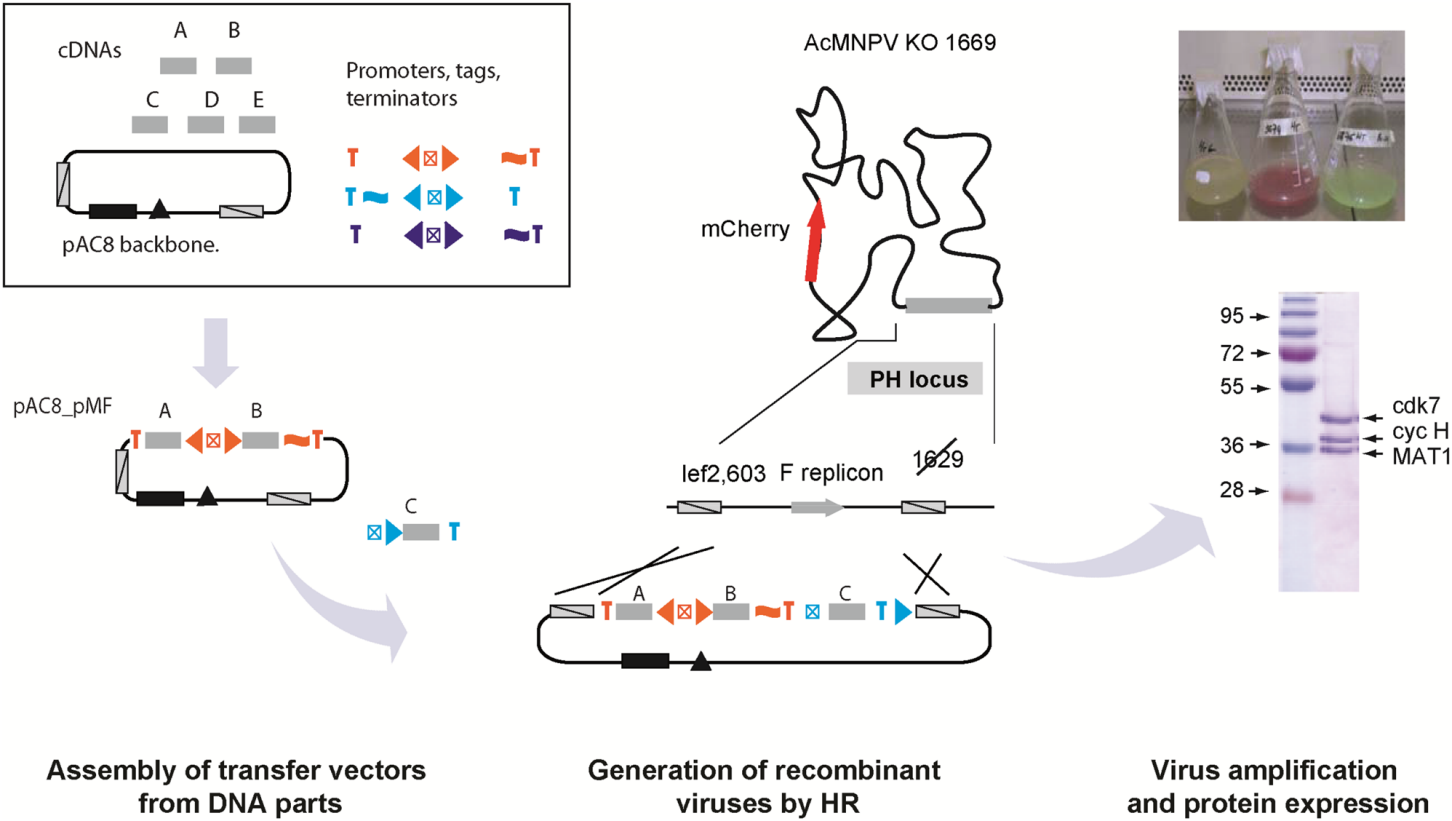
The HR-bac toolbox. To assemble dual expression cassettes into transfer vectors, DNA elements comprising the plasmid backbone, cDNAs encoding the target genes and promoter modules (typically comprising the PH and p10 promoters) are combined in a single-step four-fragment homology-based assembly reaction. Expression cassettes can be excised by digestion with a pair of restriction endonucleases or amplified by PCR and inserted via compatible restriction sites or homology-based cloning into the multiplication module of a progenitor plasmid. An example in which an expression cassette containing genes C is cloned into a plasmid containing genes A and B is shown. Viruses are generated by co-transfection of insect cells with a transfer plasmid enclosing the expression cassette and a bacmid containing a defective version of the viral genome. As the bacmid is unable to initiate virus replication unless rescued by the transfer vector, the need for post-production screening is eliminated and recombinant viruses can be directly amplified, allowing to generate viruses from transfer vectors and express proteins in 2 weeks. Expression of a fluorescent marker proteins allows to monitor virus propagation. The full-length gel of the purified complex is shown in Suppl. Fig. 7a.

Most current baculovirus multigene expression platforms rely on Tn7-mediated transposition for moving an expression cassette from a transfer vector into the baculoviral genome. This approach is perfectly justified in the case of a large multi-gene expression cassette whose assembly requires multi cloning steps and whose stability can be a concern. However, in the case of medium size binary and ternary expression cassettes which can be assembled using a single cloning step with multi-fragment cloning technologies (such as SLIC, In-Fusion™ or NEBuilder™), we experienced that generation of the virus by Tn7-mediated transposition was often more time-consuming than cloning itself. When many constructs have to be analyzed simultaneously, the selection of recombined bacmids (involving a transformation in bacteria, the selection of positive clones and the preparation of the modified bacmid before transfection) is often a time-limiting step. Here, we established a toolbox for expression screening and production of multiprotein complexes based on traditional homologous recombination (Fig. 5). The HR-bac toolbox comprises a set of bacmids for the preparation of the linearized baculoviral DNA needed to generate the recombinant viruses and a library of plasmids containing single or dual expression cassettes to assemble the multigene expression constructs.

Linearized viral DNA preparations can be purchased from various providers but the cost is not negligible, especially when a large number of constructs is to be tested. The HR-bac toolbox comprises a set of engineered bacmids that can be used for the preparation of the viral DNA. As the preparation of linearized baculoviral DNA only requires standard reagents for bacmid purification, the overall cost of virus generation is relatively low and testing many constructs simultaneously is affordable for most laboratories. To improve protein production and facilitate the monitoring of viral infection, the baculoviral cathepsin protease (*v-cath*) and chitinase (*chiA*) coding genes^18,19^ were replaced by an expression cassette for a fluorescent reporter (mCherry or EGFP). For this modification, we used lambda red recombineering and designed a targeting plasmid, which in addition to the flanking homology regions of the *v-cath* and *chiA* genes and an ampicillin selection maker contains a pair of unique restriction sites to accommodate an expression cassette. We expect this setup to be also useful for project-specific engineering of the AcMNPV genome and in particular for co-expression of helper proteins required for native post-translational modifications such as kinases, phosphatases, pro-protein convertases, and glycosylation.

As the linearized viral DNA lack an essential gene, it is unable to initiate virus replication unless rescued by the transfer vector. The need for post-production screening by FACS or traditional plaque assay is eliminated and recombinant viruses can be directly amplified, allowing to generate viruses from transfer vectors and express proteins in 2 weeks. A single round of viral amplification is typically sufficient to prepare enough virus for liter-scale productions, the time between initial cloning and protein production is shortened to 2/3 weeks (5–7 days to obtain the initial virus stock, 5–7 for virus amplification and 2–3 days for protein production).

To facilitate the assembly of multigene expression cassettes, we designed a library of single and dual expression transfer vectors loaded with N- or C-terminal affinity tags to facilitate expression screening and design of efficient purification strategies. In a typical experiment, to generate an expression cassette for co-expression of two proteins, the plasmid backbone is combined with the promoter module (comprising the PH and p10 promoters) and cDNAs encoding the two target genes, in a four-fragment restriction-free assembly reaction^25^. These plasmids possess multiplication modules and LoxP sequences which enable the rapid assembly of co-expression constructs based on tandem cloning by restriction-ligation with rare cutters and Cre/Lox recombination, respectively (Suppl. Fig. 3). Importantly, this technological framework relies on a common standardized cloning strategy modelled on the MultiBac system which therefore facilitates the exchange of DNA parts and assembly of multigene constructs.

We successfully used homologous recombination to generate a set of viruses for the production of binary and ternary complexes, suggesting that this approach constitutes an attractive alternative to Tn7-mediated transposition for medium size complexes. We are aware than the failure rate of HR-based approaches is likely to be higher than of Tn7-mediated transposition and to increase with the size of the target complex. Using several medium-size multiprotein complexes, we showed that virus pools obtained by HR-mediated recombination can be successfully used for production. The simplicity of the procedure is an important advantage, in particular to generate many expression viruses in parallel. This approach has been particularly useful for the reconstitution and functional dissection of multiprotein complexes involved in transcription regulation including the pTEFb^21^ and the transcription/DNA repair factor TFIIH^23,24,26,27^. Homologous Recombination is perhaps not the best technology to generate a few baculoviruses but is ideally suited for screening or/and expressing many constructs simultaneously when several permutations (truncations or point mutations) need to be tested: (i) HR allows to rapidly generate viruses using a single step procedure and (ii) the linearized viral DNA, often expensive when purchased from vendors, can be prepared at low cost using standard reagents. The HR-bac expression toolbox is open-source and available through http://www.addgene.com.

## Material and methods

### Engineering of the viral genome

DH10B™ cells (ThermoFisher scientific) containing the Kanamycin and Chloramphenicol resistant AcMNPV bacmid BAC10:KO_1629_^7^ were transformed with the plasmid pSC101-BAD-gbaA^28^. This Tetracycline resistant plasmid harbors mCherry encoding gene under the control of the pBAD promoter which is tightly regulated by l-arabinose and the temperature-sensitive pSC101 replicon. A single colony was used to inoculate LB media complemented with Chloramphenicol and Tetracycline. After overnight growth at 30 °C, 1:100 dilution of the culture was transferred to 100 mL of medium and once OD600 reached 0.3, l-arabinose (A3256, Sigma) was added to 0.5% w/v. After 60 min, the culture was pelleted at 4 °C, washed three times with ice-cold 10% glycerol, re-suspended in the minimal volume of ice-cold 10% glycerol, frozen in liquid nitrogen and stored at − 80 °C.

For the recombineering experiments (Suppl. Fig. 1a), a donor PCR product containing the ampicillin resistance gene and an expression cassette for a fluorescent reporter gene flanked by *chiA* and *v-cath* homology regions was amplified from the pKI shuttle vector (Suppl. Fig. 1b), by PCR using primers chia-FW and v-cath-RV. Amplification was performed with Phusion™ High-Fidelity DNA polymerase (F503, ThermoFisher scientific) with an initial denaturation step at 95 °C for 5 min, followed by 30 cycles of 95 °C for 45 s, 60 °C for 60 s and 72 °C for 2 min and a final extension step at 72 °C for 10 min. The PCR product was treated with DpnI (10 u for a 50 µL reaction), purified using a PCR-Clean Up kit and its concentration adjusted to 100 ng/µL.

For each DNA transformation, 200 ng of PCR products were added to a 50-µL aliquot of competent cells, gently mixed and then transferred to a 0.1-cm cuvette for electroporation using a BTX ECM630 electroporator (1350 V, 600 Ohms, 25 µF). After pulsing, 500 µL of LB without antibiotic was added to the cells and the suspension was incubated 90 min at 30 °C, of which 100 µL were plated onto an LB agar plate containing Chloramphenicol and Ampicillin and incubated at 37 °C. Colonies were further analyzed by PCR using the primer pairs Chia_control_FW/Amp_FW and v-cath_control_RV/mCherry_RV (Suppl. Table 3).

AcMNPV bacmids were purified using a Nucleobond PC500 DNA purification kit (740574 Macherey–Nagel) as described earlier^29^. Eluted DNA was precipitated with 0.7 volume of isopropanol, re-suspended at a concentration of 0.1 µg/µL and digested with Bsu36I enzyme (NEB) (Suppl. Fig. 1c). After enzyme was inactivated by heating at 80 °C during 20 min, aliquots were stored at − 80 °C.

### Design of modified transfer vectors

Plasmids were propagated in DH5α cells with the exception of pSPL/pUCDM derivatives, which were propagated in PIR1 bacteria. All modifications of vectors were validated by sequencing. Plasmids pAC8_GWs and pAC8_MF (Fig. 2b) were obtained from the corresponding pAC8 vectors^15^:

#### pAC8_GWs

A LoxP sequence was inserted in the unique EcoRV site of pAC8 plasmids using the set of complementary oligonucleotides LoxP_FW and LoxP_RV by restriction/ligation. Resulting plasmids were adapted to Gateway cloning by inserting the RfA conversion cassette (11828029, ThermoFisher scientific) into the PmeI site of the NdeI-BamHI linker. cDNAs inserted into Gateway Entry vectors can be transferred into the pAC8_GWs destination vectors using LR reactions. Note that the AAA-AAA triplet in the AttL1 site of the entry vector should be in phase with the reading frame of the target gene.

#### pMF-dual

A DNA fragment containing a LoxP site and dual expression cassette was amplified from pSPL with the primer pair pMF-LoxP-F/pMF-Rev and inserted between the restriction sites EcoRV and PacI of the pBacPak8 plasmid (Clontech) by restriction-ligation. The different pAC8_MF derivatives (Fig. 2b) harboring N- or C-terminal tags (10His, 6His, Flag, HA, c-myc, Twin Strep, see Suppl. Table 2) were obtained by annealing of complementary oligonucleotides or by PCR and inserted into the XhoI, NheI (p10 Nter, p10 Cter respectively) or BamHI, XbaI (PH Nter, pH Cter respectively) restriction sites, by leaving the structure of the pAC8_MF Multiple Cloning Site unchanged. cDNAs encoding the GOIs are inserted into the dual expression cassettes of pAC8_MF using the XhoI/NheI restriction sites for the gene under the control of p10 promoter and the BamHI/XbaI restriction sites for the gene under the control of pH promoter. Cloning is performed as a single step reaction based on the Sequence and Ligation Independent Cloning (SLIC) or other assembly techniques (In-Fusion, Gibson Assembly, NEBuilder). Amplified cDNAs are assembled with the promoters and the backbone regions of the plasmid isolated by restriction (XhoI/BamHI for the 5′ and XbaI/NheI for the 3′ ends of the cDNA) or amplified with primers Prom-Bam-F, Prom-Xho-R, Backbone-F, Backbone-R^25^.

#### pKI_Ø, pKI_mCherry and pKI_EGFP

The pKI shuttle vectors contain the integration cassette used to replace the *chiA* and *v-cath* genes in the baculovirus genome (Suppl. Fig. 1b). The cassette is composed of the homology arms HomA and HomB from the *chiaA* and *v-cath* genes which flank an ampicilin resistance gene and, in the case of pKI_mCherry and pKI_EGFP, the mCherry and EGFP expression units. The plasmid pKI_φ was obtained by introducing an AvrII site into the plasmid pKIloxP^13^ using PCR with the oligonucleotides pKI-FW and pKI-RV. The unique PmeI and AvrII restriction sites from pKI_ φ were used to insert the fluorescent reporter cassette obtained by digestion of pSPL_p10/mCherry and pSPL_p10/EGFP with the SpeI and PmeI restriction enzymes.

### Generation of recombinant baculoviruses and protein production

Preparation of recombinant baculoviruses and protein production was performed using established protocols^15,20,29^. In brief, 750 ng of pAC8 plasmid preparation and 250 ng linearized AcMNPV bacmid were co-transfected using 3.0 µL of Lipofectamine™ 2000 (ThermoFisher Scientific) in 35 mm dishes or individual wells of 6-well plates (9.6 cm^2^) seeded with 0.75 × 10^6^ Sf9 cells grown in Grace Insect medium supplemented with 10% FCS. As Lipofectamine transfection is inhibited by serum, we carefully adhered to the manufacturer’s instructions and performed transfection in serum free medium. After 4 h incubation, the transfection mixture was replaced by serum-supplemented medium and cells were incubated for 5–7 days at 27 °C. The supernatant constitutes the initial virus stock (V0). Viruses were amplified by adding 50 µL of V0 to 50 mL suspension cultures of Sf9 cells (1 × 10^6^ cells/mL) that were incubated for 5–7 days. Protein expression was evaluated and optimised in 6 well plates or 10 mL suspension culture prior to scale-up. For production at larger scale, suspension cultures of Sf9 or Sf21 cells (1 × 10^6^ cells/mL) cultivated in Sf900-II were infected with the appropriate volume of virus (V1) and collected 2–3 days post-infection. Cells were washed in PBS containing 10% glycerol and stored at − 80 °C.

### Baculovirus isolation and PCR analysis

#### Plaque purification

Serial dilutions of the V0 virus stock obtained from the co-transfection of the pAC8_MF_CAK transfer plasmid and the BAC10:KO_1629_, ∆v-cath/chiA, mCherry viral DNA (10^1^ to 10^6^) were seeded on plates containing 2.7 × 10^6^ Sf9 cells and incubated 1 h at 27 °C. The inoculum was removed, and infected cells were covered by medium containing 10% FCS, 40% LB medium and 50% agarose. After 5–6 days incubation at 27 °C, 24 virus-forming plaques were identified using mCherry fluorescence microscope and plugs of the overlay from the selected plaques were transferred in 1 mL of medium. Following overnight incubation at 27 °C, 60 µL of each isolate were amplified in 6-well plate seeded with 1 × 10^6^ Sf9 cells, leading to clonal virus stocks.

#### PCR analysis

Baculoviral DNA were extracted by using the Nucleospin^®^ Virus kit (740983, Marcherey-Nagel). For PCR amplification, 250 ng of viral DNA were added to a 20 µL mix containing 0.02 U/µL Phusion™ High-Fidelity DNA Polymerase (F503, ThermoFisher Scientific), 200 µM dNTP (R0192, ThermoFisher Scientific), 1X GC buffer, 3% DMSO and 0.5 µM of the primer pairs: orf 1629 F-CDK7 R (PCR1), CCNH F-CCNH-R (PCR2) or MAT1 F-Lef2, orf603 R (PCR3) (Fig. 4a and Suppl. Table 2). PCR products obtained from 4 of the tested cloned were sequenced to verify that the expected fragment was amplified.

### Production and purification of P-TEFb and XPG.

#### Expression of human P-TEFb

CDK9 fused to an N-terminal TwinStrep tag was cloned under the control of the PH promoter while N-terminal Flag-cyclin T1 was inserted under the control of p10. The CDK9/ cyclin T1 expression cassette assembled in the pKL vector from the Multibac suite^19^ was transferred into the pAC8_MF backbone using the unique PmeI and AvrII restriction sites yielding pAC8_MF-CDK9/ cyclin T1. Viruses for P-TEFb expression were generated by co-transfection of the pAC8_MF-CDK9/ cyclin T1 transfer vector with the AcMNPV BAC10:KO_1629_, Δv-cath/chiA, mCherry bacmid. The virus for P-TEFb expression obtained by Tn7-mediated transposition was previously described^21^.

#### Expression of XPG

The cDNA encoding the *Komagataella phaffii* (Pichia pastoris) XPG homologue (C4R890_KOMPG) was cloned under the control of the PH promoter of a pAC8_MF-pH-TwinStrep-Nter transfer vector already containing the mCherry cDNA yielding the pAC8_MF TwinStrep-XPG/mCherry plasmid. Viruses for XPG expression were generated by co-transfection of the transfer vector with the AcMNPV BAC10:KO_1629_, Δv-cath/chiA bacmid.

#### Purification of pTEFb and XPG

Cell pellets from infected suspension cultures (1 mL for 25 × 10^6^ cells) were disrupted by sonication in buffer A (20 mM Tris/HCl pH8, 250 mM NaCl, 0.1% NP40, 1 mM DTT and EDTA free protease inhibitor cocktail (Roche)) and the clarified lysate subjected to Strep-Tactin^®^XT affinity chromatography (IBA-Lifesciences). Proteins were eluted in the same buffer supplemented with 10 mM desthiobiotin.

### Production and purification of nuclear hormone receptor complexes

#### Expression of PPAR/RXR

The cDNA was transferred from the gateway entry vector pDONR207 into pAC8-HGWs using an LR reaction performed with the Gateway™ LR Clonase™ II enzyme mix (11791020, Invitrogen) yielding pAC8-HGWs-PPAR. The latter was fused to pSPL-RXR, a pSPL derivative^14^ comprising the gene encoding for RXR under the control of the PH promoter. For in vitro Cre/LoxP reactions 0.3 µg of each vector are mixed with 1 µL of Cre recombinase buffer 10× (500 mM Tris–HCl, 330 mM NaCl, 100 mM MgCl_2_, pH 7.5 @ 25 °C) and 1 µL of Cre recombinase enzyme prepared as described in. Reaction is performed at 37 °C during 15 min in 10 µL final volume. Reaction is stopped by heating the reaction mixture during 15 min at 70 °C, which is transformed in DH5α. For expression of PPAR and of the PPAR/RXR heterodimer, viruses were generated by co-transfection of the transfer vectors with the AcMNPV BAC10:KO_1629_, Δv-cath/chiA bacmid.

#### Purification of PPAR/RXR

Cells re-suspended in buffer B (50 mM Hepes pH7.5, 500 mM NaCl, 2 mM Chaps, 5% glycerol, 5 mM imidazole, and EDTA free protease inhibitor cocktail (Roche™)), were lysed by sonication (Vibracell sonicator, 3 mm probe at 20% intensity for 30 s (2 s ON/2 s OFF) and the clarified extract (25,000*g* for 30 min) was subjected to Ni-affinity chromatography using an HisTrap™ FF column (Cytiva). In the case of the His-tagged PPAR/RXR heterodimer, after addition of 2 molar equivalent of rosiglitazone and 9-cis-retionic acid (PPAR and RXR ligands required to stabilize the heterodimer), complex containing fractions were injected on Superdex™ 200 Increase 10/300 size exclusion column (Cytiva) equilibrated in buffer C (50 mM Hepes pH 7.5, 75 mM NaCl, 75 mM KCl, 2 mM Chaps, 5% glycerol and 2 mM TCEP).

#### Expression and purification RAR/RXR

The RAR and RXR cDNAs were inserted under the control of the PH and p10 promoters of pAC8_MF-pH-TwinStrep-Nter and the corresponding virus was generated by co-transfection with the AcMNPV BAC10:KO_1629_, Δv-cath/chiA bacmid. The complex was produced in Sf9 cells and, after addition of 2 molar equivalent of 9-cis-retionic acid (RAR and RXR ligand required to stabilize the heterodimer) the corresponding clarified extract was subjected to Strep-Tactin^®^XT affinity chromatography (IBA-Lifesciences). Proteins were eluted in the same buffer supplemented with 10 mM desthiobiotin.

### Production and purification of CAK and CAK/XPD

#### Expression of CAK and CAK/XPD

The genes coding for the CDK7 kinase, cyclin H, and MAT1 were cloned in pAC8_MF. The organization of the promoter cassette in pAC8_MF is identical to that of the pAcAB3 triple expression vector^30^ with ORF coding for CDK7 cloned under the control of p10 promoter, and ORFs coding for cyclin H and MAT1 proteins cloned under the control of PH promoters but in opposite orientation. The expression levels of cyclin H and MAT1 being higher than that of CDK7 (Suppl. Fig. 5b), the kinase was cloned with a C-terminal strep tag to ensure stochiometric amounts of each subunit after purification. The cDNA encoding full length XPD fused to an N-terminal Flag sequence was inserted under the control of the PH promoter in the pSPL_p10/DsRed donor vector and the resulting vector fused to the pCA8_MF-CAK acceptor plasmid using in vitro Cre-mediated recombination as described above. Viruses were generated by co-transfection of the transfer vectors with the AcMNPV BAC10:KO_1629_, Δv-cath/chiA bacmids.

#### Purification of CAK and CAK/XPD

For purification of CAK, a cell pellet from an infected suspension culture is disrupted by sonication in buffer A (20 mM Tris/HCl pH8, 250 mM NaCl, 0.1% NP40, 1 mM DTT and EDTA free protease inhibitor cocktail (Roche)) and the clarified lysate subjected to Strep-Tactin^®^XT affinity chromatography (IBA-Lifesciences). Proteins were eluted in the same buffer supplemented with 10 mM desthiobiotin. For purification of the CAK/XPD complex cells were disrupted by sonication in buffer A, incubated with Anti Flag^®^M2 affinity gel (Sigma-Aldrich) using 1 mL of resin for 1 L of culture for 2 h at 4 °C with gentle shaking. After extensive washes in buffer D (20 mM Tris–HCl pH 7.5, 250 mM NaCl), bound proteins were eluted with the same buffer containing 1 mg/mL Flag peptide (sequence: DYKDDDDK) and further subjected to Strep-Tactin^®^XT affinity chromatography (IBA-Lifesciences).

## Supplementary Information

Supplementary Information.
