# Author Correction: HR-Bac, a toolbox based on homologous recombination for expression, screening and production of multiprotein complexes using the baculovirus expression system

**DOI:** 10.1038/s41598-022-07495-8

**Published:** 2022-02-24

**Authors:** Olga Kolesnikova, Amélie Zachayus, Simon Pichard, Judit Osz, Natacha Rochel, Paola Rossolillo, Isabelle Kolb-Cheynel, Nathalie Troffer-Charlier, Emmanuel Compe, Olivier Bensaude, Imre Berger, Arnaud Poterszman

**Affiliations:** 1 Institute of Genetics and of Molecular and Cellular Biology (IGBMC), 1 rue Laurent Fries, Illkirch, France; 2 Centre National de la Recherche Scientifique (CNRS), UMR 7104, Illkirch, France; 3 Institut National de la Santé et de la Recherche Médicale (Inserm), U964, Illkirch, France; 4 Université de Strasbourg, Illkirch, France; 5 Institut de Biologie de l’Ecole Normale Supérieure (IBENS), Ecole Normale Supérieure, CNRS, INSERM, PSL Research University, 46 rue d’Ulm, 75005 Paris, France; 6 Max Planck Bristol Centre for Minimal Biology, Cantock’s Close, University of Bristol, Bristol, BS8 1TS UK; 7 Bristol Synthetic Biology Centre BrisSynBio, School of Biochemistry, 1 Tankard’s Close, University of Bristol, Bristol, BS8 1TD UK; 8 Present Address: EMBL, Heidelberg, Germany

Correction to: *Scientific Reports* 10.1038/s41598-021-04715-5, published online 7 February 2022

The original version of this Article contained an error in the spelling of the authors Olga Kolesnikova, Amélie Zachayus, Simon Pichard, Judit Osz, Natacha Rochel, Paola Rossolillo, Isabelle Kolb-Cheynel, Nathalie Troffer-Charlier, Emmanuel Compe, Olivier Bensaude, Imre Berger, Arnaud Poterszman which were incorrectly given as Kolesnikova Olga, Zachayus Amélie, Pichard Simon, Osz Judit, Rochel Natacha, Rossolillo Paola, Kolb-Cheynel Isabelle, Troffer-Charlier Nathalie, Compe Emmanuel, Bensaude Olivier, Berger Imre, Poterszman Arnaud. The original Article has been corrected.

