# Supplementary material for: HR-Bac, a toolbox based on homologous recombination for expression, screening and production of multiprotein complexes using the baculovirus expression system
Source: Sci Rep. 2022 Feb 7;12:2030. doi: 10.1038/s41598-021-04715-5 (PMC8821708; doi:10.1038/s41598-021-04715-5)
Supplement: Supplementary file 1 — Supplementary Information. [file 41598_2021_4715_MOESM1_ESM.pdf]

Supplementary figures and tables for:

HR-Bac, a toolbox based on homologous recombination for expression screening and production of multiprotein complexes using the baculovirus expression system.

Kolesnikova Olga[1,2,3,4,#], Zachayus Amélie [1,2,3,4], Pichard Simon[1,2,3,4], Osz Judit[1,2,3,4], Rochel Natacha[1,2,3,4] Rossolillo Paola[1,2,3,4], Kolb-Cheynel Isabelle[1,2,3,4], Troffer-Charlier Nathalie[1,2,3,4], Compe Emmanuel[1,2,3,4], Olivier Bensaude[5], Berger Imre[6], Poterszman Arnaud[1,2,3,4,*]

[1] Institute of Genetics and of Molecular and Cellular Biology (IGBMC), 1 rue Laurent Fries, Illkirch, France.

[2] Centre National de la Recherche Scientifique (CNRS), UMR 7104, Illkirch, France.

[3] Institut National de la Santé et de la Recherche Médicale (Inserm), U964, Illkirch, France.

[4] Université de Strasbourg, Illkirch, France.

[5] Institut de Biologie de l'Ecole Normale Supérieure (IBENS), Ecole Normale Supérieure, CNRS, INSERM, PSL Research University, 46 rue d'Ulm, 75005 Paris, France

[6] Max Planck Bristol Centre for Minimal Biology, Cantock's Close, University of Bristol, Bristol BS8 1TS, Bristol Synthetic Biology Centre BrisSynBio, School of Biochemistry, 1 Tankard's Close, University of Bristol, Bristol BS8 1TD, UK

[#] at present: EMBL, Heidelberg

[*] Corresponding author (Arnaud.Poterszman@igbmc.fr)

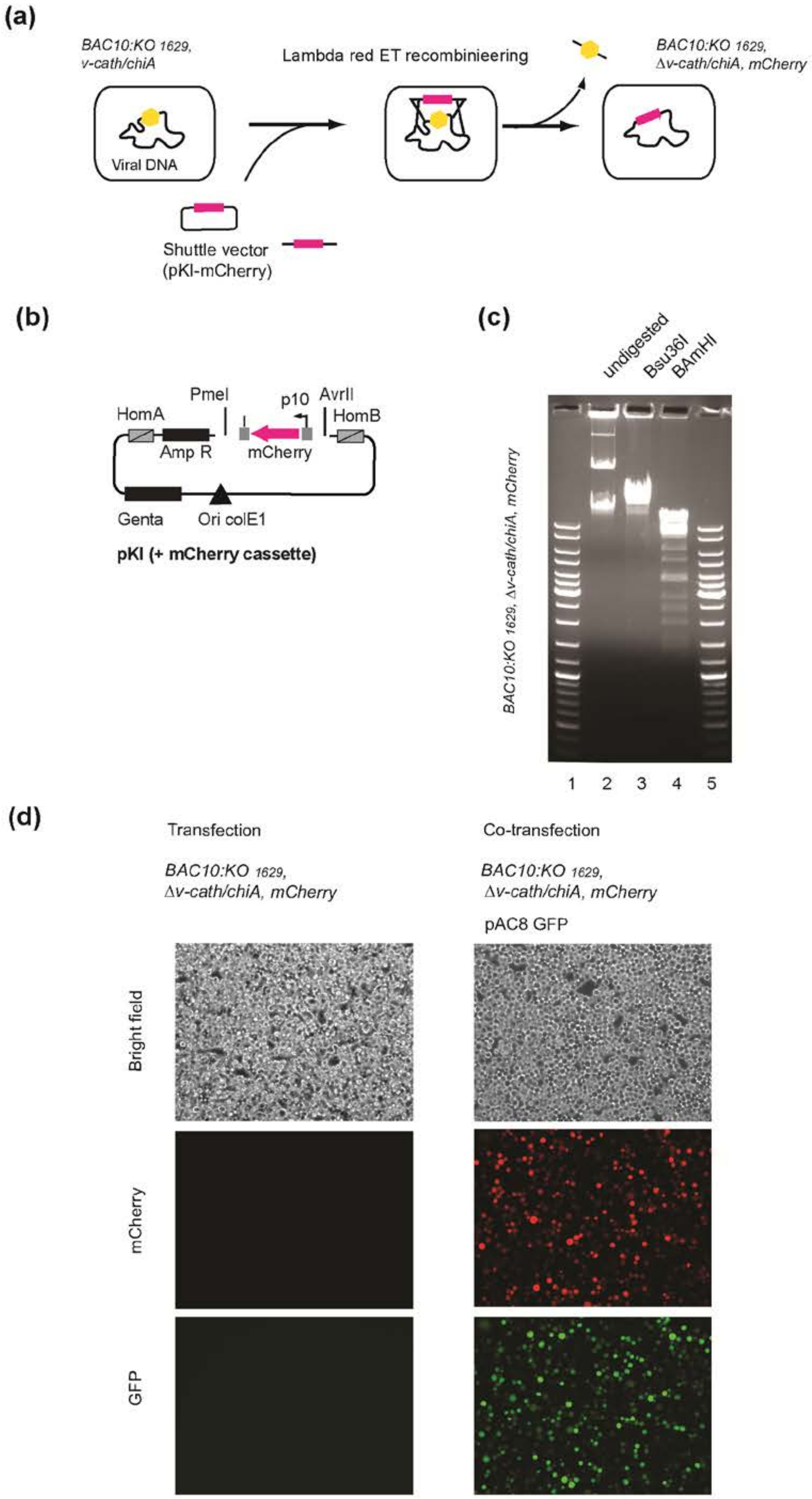
(a)
BAC10:KO 1629,
v-cath/chiA
Lambda red ET recombinieering
BAC10:KO 1629,
Δv-cath/chiA, mCherry
Viral DNA
Shuttle vector
(pKI-mCherry)
(b)
PmeI
p10
AvrII
HomA
HomB
Amp R
mCherry
Genta
Ori colE1
pKI (+ mCherry cassette)
(c)
undigested
Bsu36I
BAmHI
BAC10:KO 1629, Δv-cath/chiA, mCherry
1
2
3
4
5
(d)
Transfection
Co-transfection
BAC10:KO 1629,
Δv-cath/chiA, mCherry
BAC10:KO 1629,
Δv-cath/chiA, mCherry
pAC8 GFP
Bright field
mCherry
GFP

Supplementary Figure 1. **Generation of BAC10:KO$_{1629}$ Δv-cath/chiA,mCherry DNA using λ-Red recombineering. (a)** Schematic representation of the workflow. *E. coli* cells bearing viral DNA AcMNPV BAC10:KO$_{1629}$ in a form of a bacmid were transformed with a linear fragment composed of the mCherry coding sequence under the control of the p10 promoter (red rectangle) flanked with sequences homologous to *v-cath* and *chiA* flanking regions. Induction of λ-Red recombineering resulted in the replacement of *v-cath* and *chiA* coding region in baculoviral DNA (yellow hexagon) by mCherry coding sequence. **(b)** Schematic representation of the pKI-mCherry shuttle plasmid where the mCherry cDNA is inserted downstream the p10 promoter. In pKI_EGFP, the cDNA encoding mCherry is replaced by the EGFP coding sequence. In pKI_ϕ, the PmeI/ArvII fragment was deleted. **(c)** Restriction analysis of purified DNA AcMNPV BAC10:KO$_{1629}$, Δv-cath/chiA, mCherry. Ethidium bromide–stained 0.8% agarose gel representing purified bacmid DNA (lane2) digested with Bsu36I or BamHI (lanes 3 and 4, respectively). Molecular weight markers have been loaded in lanes 1 and 5. **(d)** Micrographs of Sf9 cells co-transfected with AcMNPV BAC10:KO$_{1629}$ Δv-*cath/chiA*,mCherry DNA and the pAC8-His-EGFP transfer plasmid for EGFP expression (right panel) or transfected with AcMNPV BAC10:KO$_{1629}$ Δv-*cath/chiA*,mCherry DNA in absence of pAC8-His-EGFP (left panel). Cells were incubated at 27°C for 5 days and the specific fluorescence signals of mCherry and EGFP for the same field were observed using an inverted microscope with a 10x objective.

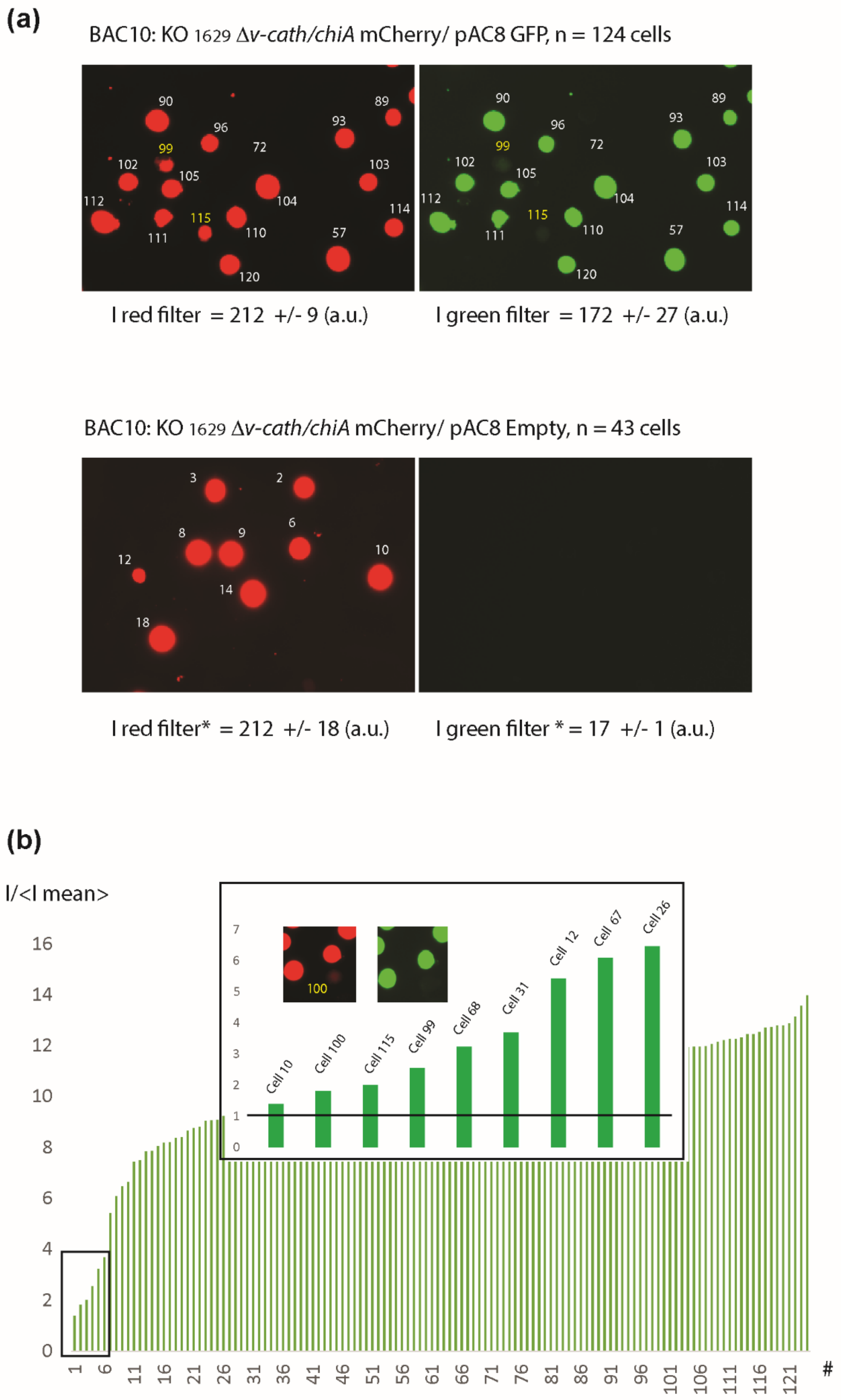
(a)
BAC10: KO 1629 Δv-cath/chiA mCherry/ pAC8 GFP, n = 124 cells
I red filter = 212 +/- 9 (a.u.)
I green filter = 172 +/- 27 (a.u.)
BAC10: KO 1629 Δv-cath/chiA mCherry/ pAC8 Empty, n = 43 cells
I red filter* = 212 +/- 18 (a.u.)
I green filter * = 17 +/- 1 (a.u.)
(b)
I/<I mean>
Cell 10
Cell 100
Cell 115
Cell 99
Cell 68
Cell 31
Cell 12
Cell 67
Cell 26
#

Supplementary Figure 2. Single cell analysis of Sf9 cells infected with a recombinant virus generated using AcMNPV BAC10:KO_{1629} Δv-*cath/chiA*,mCherry DNA and a transfer vector containing the EGFP cDNA. (a) Sf9 cells seeded in a 6-well plate were infected with the initial virus stock obtained by co-transfection of BAC10:KO_{1629} Δv-*cath/chiA*,mCherry DNA and the pAC8-His-GFP control plasmid. After 48 hours incubation, the specific fluorescence signals of mCherry and EGFP were observed with a 20x objective. As control which does not express EGFP, cells were infected with a virus generated with AcMNPV BAC10:KO_{1629} Δv-*cath/chiA*,mCherry DNA and an empty version of pAC8-His. **(b)** Histogram showing the normalized mean EGFP fluorescence ($I/\langle I_{mean} \rangle$ where $\langle I_{mean} \rangle$ is the mean signal measured for cells infected with a virus generated with an empty transfer vector) emitted by infected cells (#). We used automated thresholding to identify cells expressing mCherry, generated a mask and measured the specific fluorescence signals of mCherry and EGFP (Fidji version 1.52p). As judged by fluorescence intensity, cells expressing low levels of EGFP also express limited amounts of mCherry (see cell #100 which is barely visible in the insert).

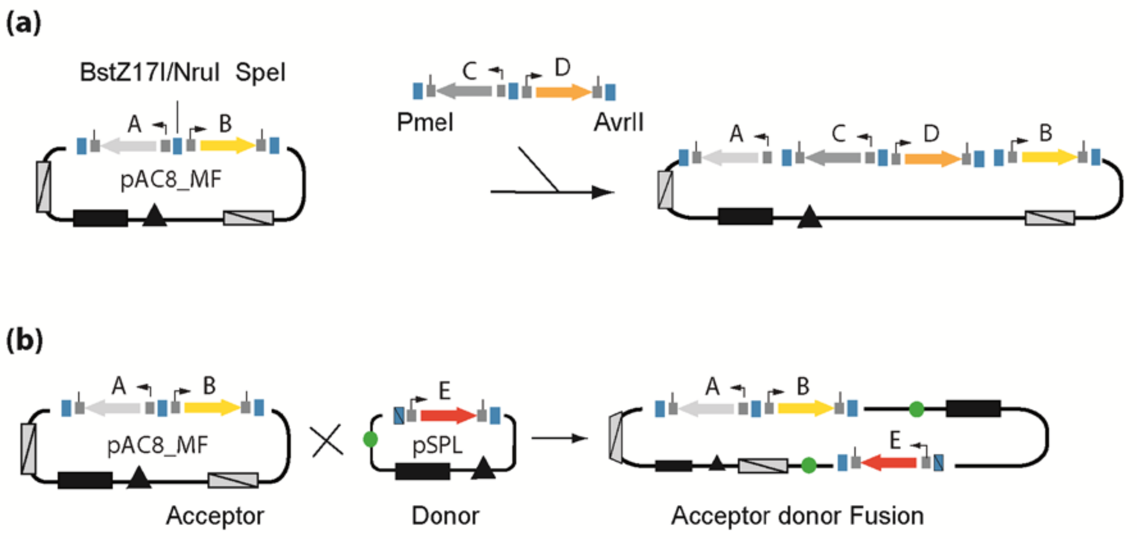

Supplementary Figure 3. Assembly of multigene expression vector from pre-assembled plasmids (a) Concatenation of dual expression cassettes by restriction ligation. A dual expression cassette obtained by digestion of a donor plasmid using the PmeI/AvrII restriction sites is ligated into an acceptor pAC8_MF vector linearized using the BstZI17/SpeI or NruI/SpeI. **(b)** Schematic representation of the Cre-mediated plasmid fusion between the acceptor pAC8_MF and a pSPL donor. Lox P sites are represented by green circles.

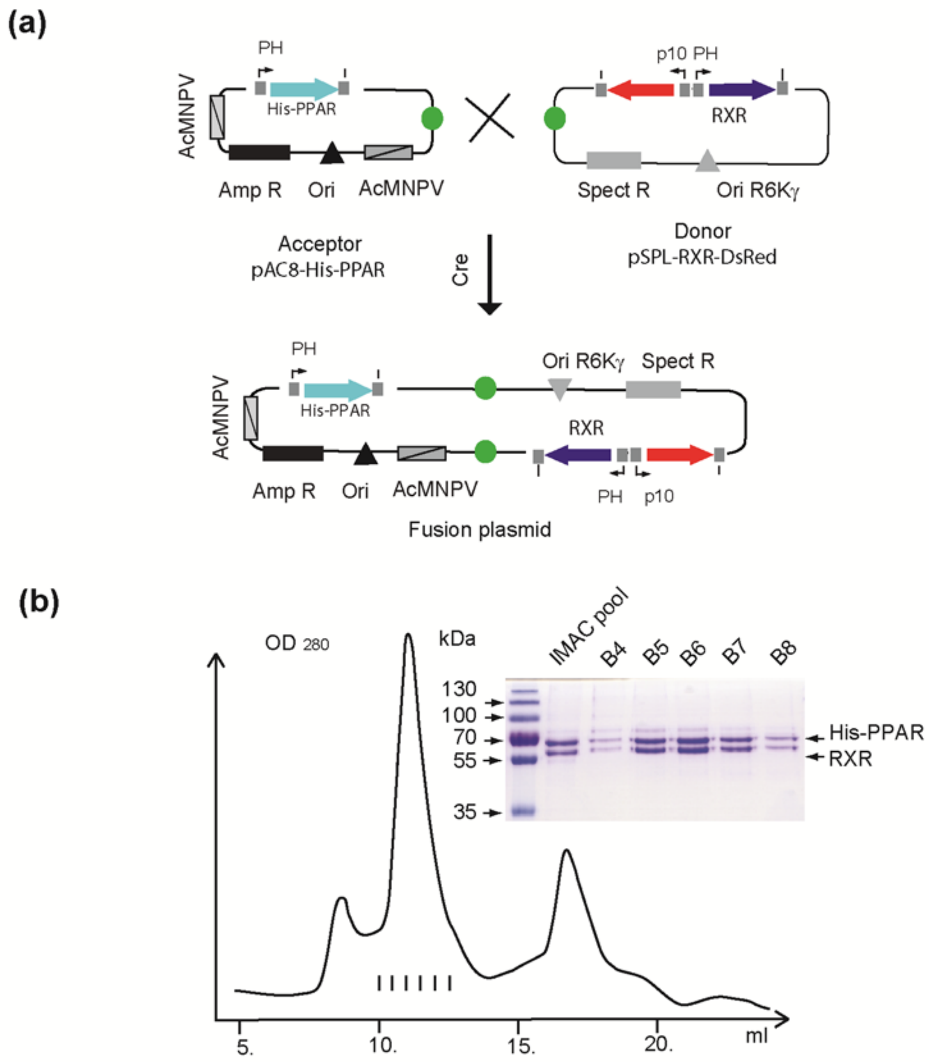

Supplementary Figure 4. Production of the PPAR/RXR heterodimer. (a) Schematic representation of the Cre-mediated plasmid fusion between the acceptor pAC8-His-PPAR and the donor pSPL-RXR-DsRed. Lox P sites are represented by green circles. **(b)** The histidine tagged PPAR/RXR heterodimer produced using a virus obtained from the pAC8-His-PPAR/pSPL-RXR-DsRed construct (co-transfected with the AcMNPV BAC10:KO1629, Δv-cath/chiA DNA bacmid) was purified by Ni-affinity chromatography. Fractions containing the complex were loaded on a Superdex™ 200 Increase 10/300 column and selected peak fractions were analysed on a Coomassie-stained 12.5% SDS gel (shown in the insert). Full-length version of the gel from panel 4b is shown in Suppl. Fig. 7b.

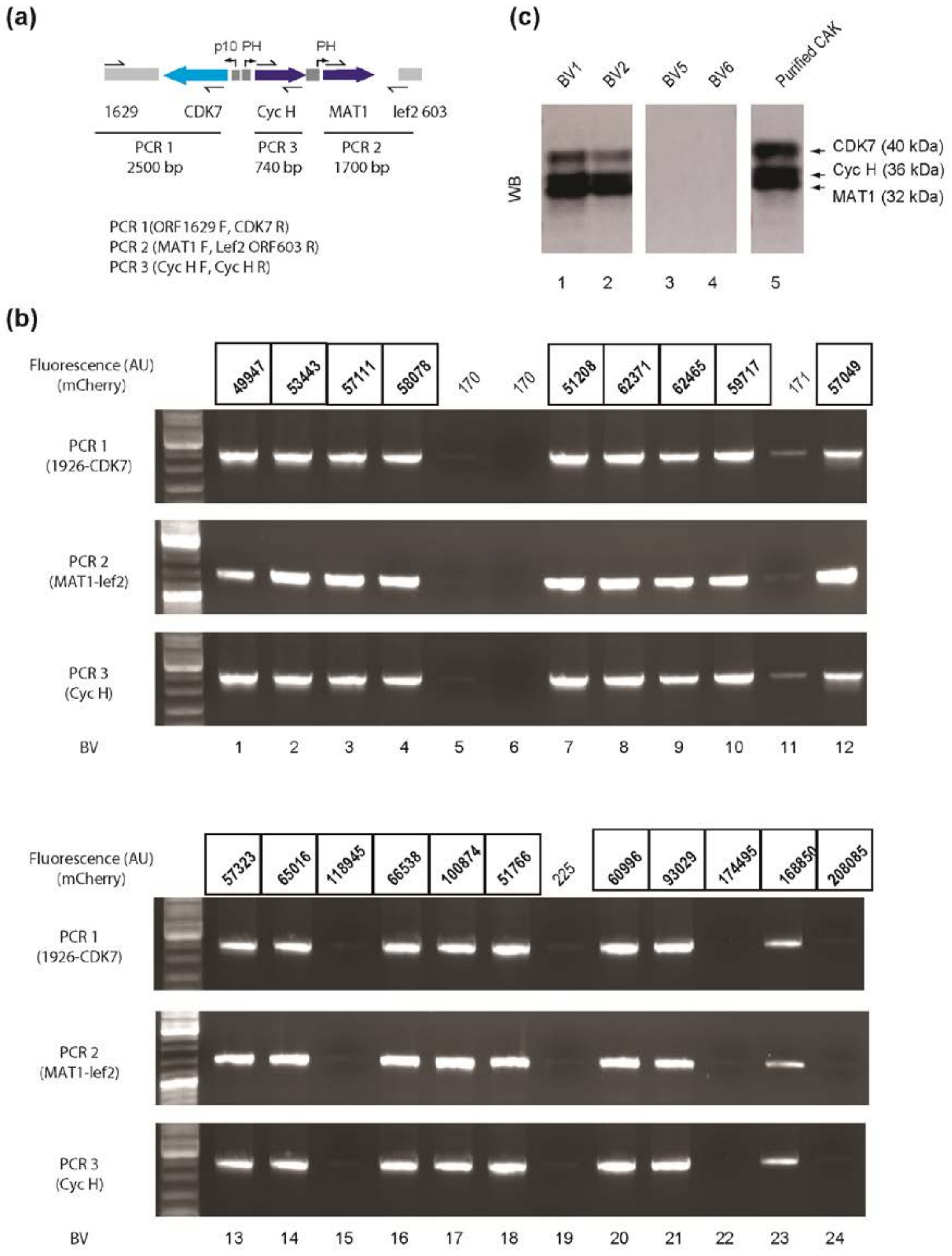

Supplementary Figure 5. **Analysis of a virus pool: (a)** Organization of the CAK expression cassette. cDNAs encoding Cdk7, cyclin H and MAT1 assembled into pAC8_MF. Primer pairs used for the PCR analysis of virus integrity are listed. **(b)** Baculoviral clones from a CAK expressing virus pool obtained using the BAC10:KO$_{1629}$, Δv-cath/chiA, mCherry viral DNA were

isolated using plaque purification and amplified. mCherry fluorescence was measured to identify clones that were successfully amplified. In parallel, the viral DNA was extracted and the integrity of the CAK expression cassette was analyzed by PCR: 17 out of the 20 viruses that were amplified (boxed fluorescence intensities) have the expected complete structure. **(c)** Whole cell extracts prepared from Sf21 cells infected with either positive (BV1 and BV2, lanes 1-2) or negative (BV5 and BV6, lanes 3-4) baculoviral clones were resolved on 12.5% SDS PAGE and the presence of the CAK subunits was detected by Western Blot using a mixture of monoclonal antibodies raised against CDK7, Cyclin H and MAT1 (@CDK7 1/1000 2F8, IGBMC; @Cyclin H 1/1000 2D4, IGBMC and @MAT1 1/1000 2D8 and 1G6, IGBMC). A sample of purified CAK where the three subunits are present in stochiometric amounts was used as control. Full-length gels are shown in Suppl. Fig. 7c-f.

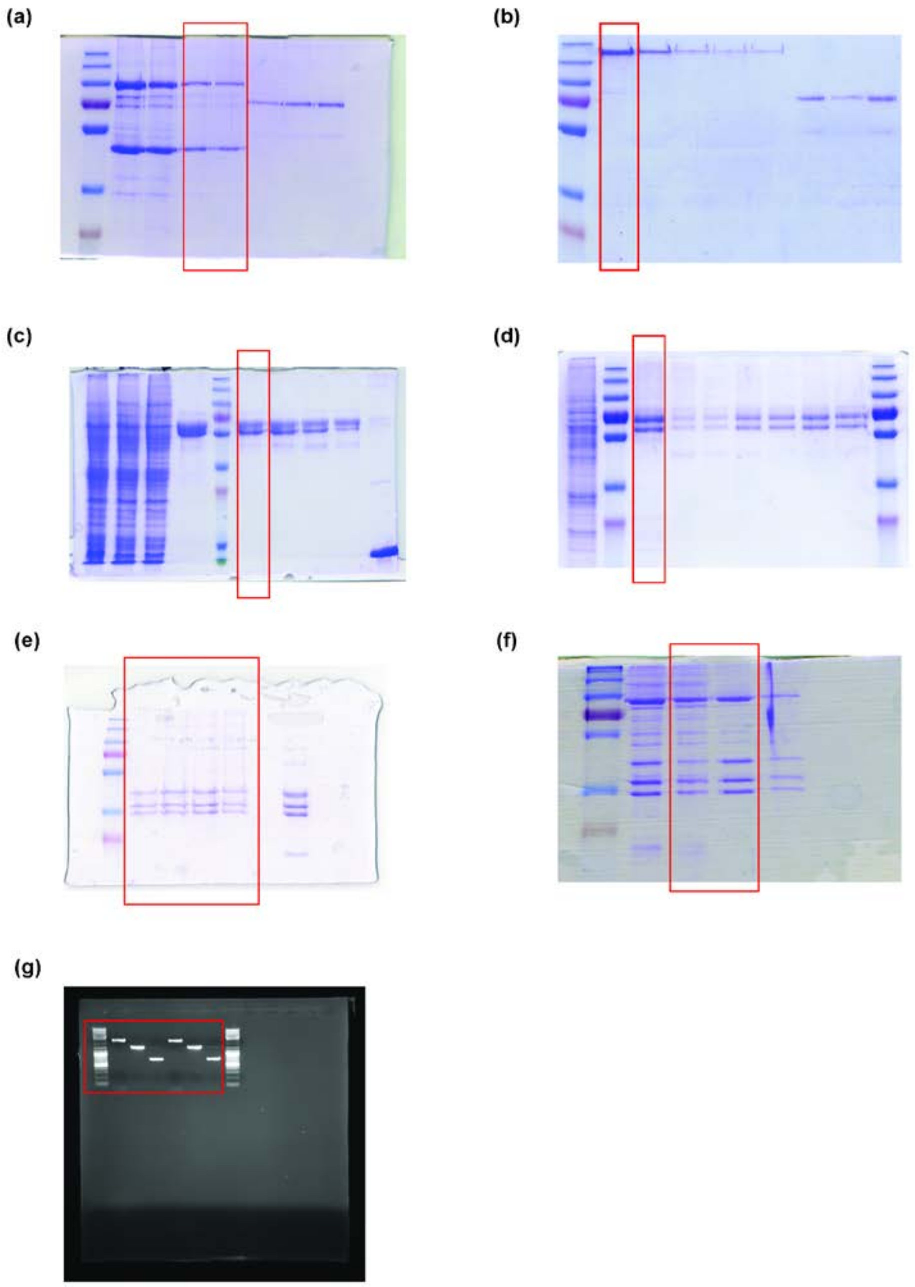

Supplementary Figure 6. **Un-cropped gels from Figures 3 and 4 where the parts shown are boxed: (a)** Cdk9/cyclin T binary complex shown in Fig. 3a (SDS-PAGE), **(b)** the XPG protein shown in Fig. 3b (SDS-PAGE), **(c, d)** RAR/RXR and PPAR/RXR heterodimers shown in Fig. 3c and 3d (SDS-PAGE), **(e, f)** CAK and CAK/XPD ternary and quaternary complexes shown in Fig. 4b and 4d (SDS-PAGE). **(g)** PCR analysis of viruses (Agarose gel) shown in Fig. 4c.

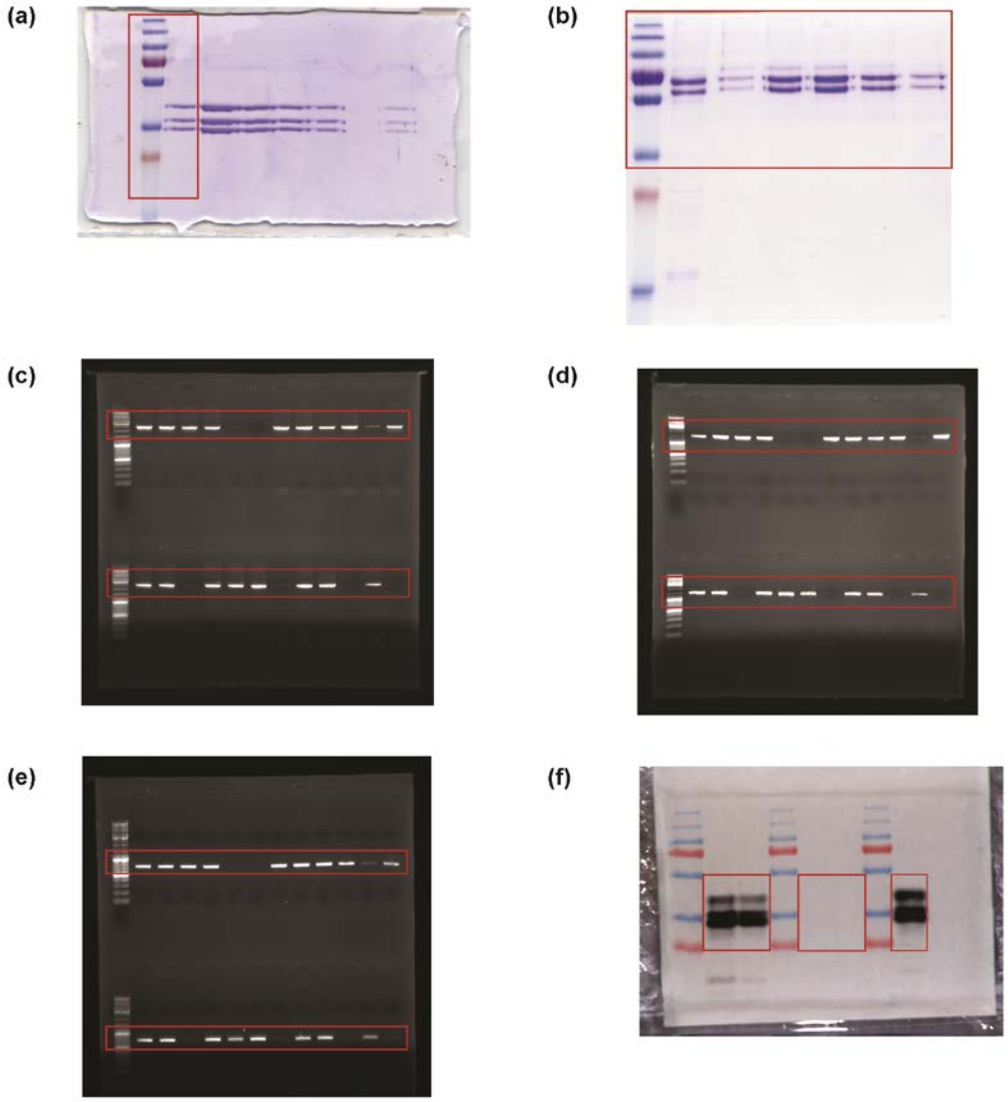

Supplementary Figure 7. **Un-cropped gels from Figures 5 and Supplementary Figures 4 and 5 where the parts shown are boxed: (a)** CAK ternary complex shown in Fig. 5 (SDS-PAGE). **(b)** PPAR/RXR heterodimer shown in Fig. 4b (SDS-PAGE). **(c, d and e)** PCR analysis of CAK expression cassettes from plaque purified viruses shown in Suppl. Fig. 5b: PCR products PCR1 (orf1629 – CDK7), PCR2 (MAT1-lef2, orf603) and PCR3 (Cyc H) are presented in panels b, c and d. **(f)** Western Blot analysis of whole cell extracts shown in Suppl. Fig. 5c.

Plasmid	Affinity tags
**pAC8-0GW**	
**pAC8-6His-3C-GW**	Nter 6xHis tag, optional Cter c-myc
**pAC8-10His-3C-GW**	Nter 10xHis tag, optional Cter c-myc
**pAC8-Flag-3C-GW**	Nter Flag tag, optional Cter c-myc
**pAC8-GST-3C-GW**	Nter GST tag, optional Cter c-myc

Supplementary Table 1. pAC8_GW transfer vector series loaded with N-terminal (Nter) and C- terminal (Cter) affinity tags.

Plasmid	p10 promoter	PH promoter
**pAC8_MF-pH-3C-10His-Cter**	-	Cter cleavable 10xHis tag
**pAC8_MF-pH-6His-Cter**	-	Cter 6xHis tag
**pAC8_MF-p10-10His-Cter**	Cter 10xHis tag	
**pAC8_MF-pH-TwinStrep-Nter**	-	Nter Twin-Strep tag
**pAC8_MF-pH-TwinStrep-Cter**	-	Cter Twin-Strep tag
**pAC8_MF-pH-Flag-Nter**	-	Nter Flag tag
**pAC8_MF-pH-Flag-Cter**	-	Cter Flag tag
**pAC8_MF-p10-Flag-Nter**	Nter Flag tag	-
**pAC8_MF-p10-Flag-Cter**	Cter Flag tag	-
**pAC8_MF-pH-HA-Nter**	-	Nter Hemagglutinin tag
**pAC8_MF-pH-HA-Cter**	-	Cter Hemagglutinin tag
**pAC8_MF-p10-HA-Cter**	Cter Hemagglutinin tag	-
**pAC8_MF-pH-cmyc-Cter**	-	Cter Myc tag

Supplementary Table 2. pAC8_MF transfer vectors loaded with N-terminal (N-ter) or C-terminal (C-ter) affinity tags.

Primer name	Primer Sequence
chia-FW (COM1399)	GAAAAATCCGTCCTCTCCCCAATCCGTG
v-cath-RV (COM1400)	ACAGAGTCGACGCTGTCAAACGAAATCAAA
chia_control_FW (COM 943)	AAACTGTGCGTTTATCGCGTTGAGCAAGT
Amp-FW	CAACATTTCCGTGTCGCCCTTATTCCC
v-cath_control-RV (COM 944)	CTTGAACTCACAACCTTAGCAAGATCTA
mCherry-RV	CTTGTACAGCTCGTCCATGCC
LoxP-FW	ATAACTTCGTATAGCATACATTATACGAAGTTAT
LoxP-RV	ATAACTTCGTATAATGTATGCTATACGAAGTTAT
pMF-LoxP-F:	GTTGCTGATATCATAACTTCGTATAGCATAC
pMF-Rev:	CAATTAATCGCCTAGGGGTTATGATAG
pKI-FW	GGCTTCGAACGCGGTACCtagGCATGCTAT
pKI-RV	ATAGCATGCctaGGTACCGCGTTCGAAGCC
PH-FW (bac1)	ACCATCTCGCAAATAAATAA
SV40-polyA	TGGTATGGCTGATTATGATC
p10-FW	CCCAACACAATATATTATAG
HSV TK polyA-RV	CACCCGTGCGTTTTATTCTGTC
orf1629 F	GTCTCGCCACTACAATGCTGTAGT
CDK7 R	GCTCTGGACGTGAAGTCTC
MAT1 F	CCTCGGTGTAAGACCACCAAATATCG
Lef2, orf603 R	GGCAACTGCAAGGGTCTCAATC
CCNH F	GCCAAGATCTGTTGTGGGTACG
CCNH R	TCTACCAGGTCGTCATCAGTCC

Supplementary Table 3. List of primers.
